# The Use of TheraBracelet Upper Extremity Vibrotactile Stimulation in a Child with Cerebral Palsy—A Case Report

**DOI:** 10.3390/electronics13163147

**Published:** 2024-08-09

**Authors:** Na Jin Seo, Molly Brinkhoff, Savannah Fredendall, Patricia Coker-Bolt, Kelly McGloon, Elizabeth Humanitzki

**Affiliations:** 1 Department of Rehabilitation Sciences, Medical University of South Carolina, Charleston, SC 29425;; 2 The Therapy Place, Columbia, SC 29204; 3 Department of Health Sciences and Research, Medical University of South Carolina, Charleston, SC 29425;

**Keywords:** wearable device, vibrotactile stimulation, stochastic resonance, repetitive peripheral sensory stimulation, subliminal stimulation, rehabilitation, upper extremity, paresis, cerebral palsy, feasibility

## Abstract

**Background:**

TheraBracelet is peripheral vibrotactile stimulation applied to the affected upper extremity via a wristwatch-like wearable device during daily activities and therapy to improve upper limb function. The objective of this study was to examine feasibility of using TheraBracelet for a child with hemiplegic cerebral palsy.

**Methods:**

A nine-year-old male with cerebral palsy was provided with TheraBracelet to use during daily activities in the home and community settings for 1.5 years while receiving standard care physical/occupational therapy.

**Results:**

The child used TheraBracelet independently and consistently except during summer vacations and elbow-to-wrist orthotic use from growth spurt-related contracture. The use of TheraBracelet did not impede or prevent participation in daily activities. No study-related adverse events were reported by the therapist, child, or parent.

**Conclusion:**

Future research is warranted to investigate TheraBracelet as a propitious therapeutic device with focus on potential impact of use to improve the affected upper limb function in daily activities in children with hemiplegic cerebral palsy.

## 1. Introduction

Cerebral palsy (CP) is one of the most common physical disabilities in childhood affecting 1 in 345 children [1]. Children with hemiplegic CP experience delayed developmental milestones, including difficulty performing age-appropriate functional activities [2]. Overall, 83% of children with CP have upper limb involvement [3]. Upper extremity hemiparesis negatively impacts an individual’s ability to be independent in self-care tasks, decreases social well-being, and diminishes their quality of life [4]. Standard care includes physical and occupational therapy but impairment typically persists throughout life [5]. Therapy services are time intensive and costly. The lifetime cost of care for individuals was over $1 million dollars back in 2003 [6].

It is critical therefore that we develop means to augment therapy enhancing functional, developmental outcomes. One such means could be peripheral sensory stimulation [7]. The scientific rationale for peripheral sensory stimulation is that afferent input can directly influence the motor cortex via direct projections from the cortical sensory to motor areas. The direct projections have been demonstrated in intracortical microstimulation [8–11] and long-latency cutaneomuscular reflex studies [12, 13]. As such, peripheral sensory stimulation can be used to prime the motor pathway in the central nervous system. Changes in the corticospinal excitability measured using transcranial magnetic stimulation (TMS) [14–17] and changes in the primary motor cortex activity measured using functional magnetic resonance imaging (fMRI) [18] and electroencephalogram (EEG) [19, 20] have been shown with peripheral sensory stimulation. Leveraging this priming effect, peripheral sensory stimulation has been used as a therapy adjuvant to augment neuroplasticity and motor function in patients with neurologic movement disorders in a number of studies [21–38]. In addition to individual randomized controlled trials, meta-analysis demonstrates that addition of peripheral sensory stimulation to therapy enhances motor function more than therapy alone [34]. However, most studies were conducted for adults who survived a stroke. Investigation for use of peripheral sensory stimulation is scarce in pediatric stroke or cerebral palsy literature.

Most modalities of peripheral sensory stimulation involve suprathreshold stimulation that causes tingling sensation [17, 18] irrelevant to tasks at hand or wear of a glove that may interfere with cutaneous feedback necessary for dexterous finger movement control [39, 40]. Therefore, most sensory stimulation modalities are administered immediately prior to therapy, requiring additional time commitment. These constraints make it difficult for patient adherence and implementation, especially for children [41]. A recently developed sensory stimulation modality, named TheraBracelet, aims to mitigate this practical limitation [36, 37]. TheraBracelet is a peripheral vibrotactile sensory stimulation applied via a device worn on the affected wrist like a wristwatch [37, 42]. TheraBracelet utilizes random-frequency subthreshold (i.e., imperceptible) vibration for stochastic resonance [43–45] to increase brain activity for the hemiparetic upper extremity [19, 20, 46, 47]. Although imperceptible, it can activate mechanoreceptors in the skin and their afferents [48, 49] as well as the upstream sensorimotor cortex [19, 20, 46] to influence sensorimotor performance [43, 50–53].

TheraBracelet has previously been used to improve the motion of the hemiparetic upper extremity in adult chronic stroke survivors [36–38]. Results from a pilot double blinded 2-week task practice therapy program showed improvement in upper extremity function greater than with therapy alone [36]. This greater improvement in the affected upper extremity function for the treatment group was accompanied by increased neural communication in the sensorimotor cortex in a longitudinal electroencephalogram study [19]. A longer 6-week program showed continued functional improvement over the 6 weeks [38]. Encouraged by these pilot studies, an adequately powered double-blind randomized controlled trial is currently in progress to investigate the clinical utility of using TheraBracelet during therapy sessions in adult stroke survivors [54].

Further, use of TheraBracelet in the home and community setting, outside of therapy sessions in the clinic or lab, could substantially increase the treatment duration. In a pilot double-blind randomized controlled trial, adult stroke survivors who received TheraBracelet stimulation from the wrist-worn device improved their upper extremity function commensurate with the level of adherence to home exercises over the 4-week period [37]. In contrast, those who received no stimulation from the device did not improve upper extremity function.

While use of TheraBracelet has been investigated for adult stroke survivors, its use has not been investigated for children with hemiplegic cerebral palsy. The wearable and imperceptible nature of the TheraBracelet stimulation affords the portability and accessibility without interference of daily routines of children who need to balance therapy with academic responsibilities. Use of TheraBracelet in the home and community settings enables maximal intervention dosage, not limited to therapy sessions. Previous studies performed in adult stroke survivors showed that the effect is pronounced in the hand-object manipulation abilities [36–38, 55]. Therefore, TheraBracelet presents a non-invasive means for potentially enhancing neural activity for the upper extremity sensorimotor hand-object manipulation tasks [19, 20, 46] in children with cerebral palsy. This type of technology could present an effective means of enhancing functional gains toward facilitating developmental milestones in children facing potential lifelong rehabilitation needs. Therefore, as the first step toward this investigation, the objective of this case report was to examine the feasibility of using TheraBracelet for the hemiplegic upper extremity in a child with cerebral palsy in day-to-day activities in the home and community.

## 2. Materials and Methods

### Participant

The participant in this case report was a nine-year-old male with right-sided hemiparesis due to cerebral palsy secondary to a stroke at birth. A clinical profile using the CP Functional Classification System are provided below

- Manual Ability Classification System [56]: Level III, he was able to handle objects with difficulty and needed help to prepare and/or modify activities. The child did not take any medications for spasticity, and he did not have a history of any movement disorders outside of hemiplegia.
- Gross Motor Function Classification System [56]: Level 1, he was able to ambulate without limitations, not requiring the use of a mobility device.
- Eating and Drinking Ability Classification Scale [57]: Level 1, he was able to eat and drink safely and efficiently.
- Communication Function Classification System [56]: Level I, he was an effective sender and receiver with unfamiliar and familiar partners.
- Visual Function Classification System [58]: Level 1, he used visual functions easily without compensatory strategies.

These scores indicate that overall, he had a high level of functioning in most areas but had the most difficulty using his arm and hand to interact with items in his environment. This child therefore was a great case study example focusing on potential functional gains that could be made relative to his area of greatest impairment.

He and his parent were interested in participating in this study to gain more mobility and functional ability of his right hemiparetic hand and digits. The child received occupational therapy twice a week, physical therapy once a week, and summer hippotherapy while participating in this study. The child attended school in the 3^rd^ and 4^th^ grades during the study period and did not receive any school-based therapy services. This study was approved by the Medical University of South Carolina Institutional Review Board. The child’s parent signed a consent form prior to his participation in the study.

### Procedure

#### TheraBracelet Wearable and Smartphone App

**Figure 1.**
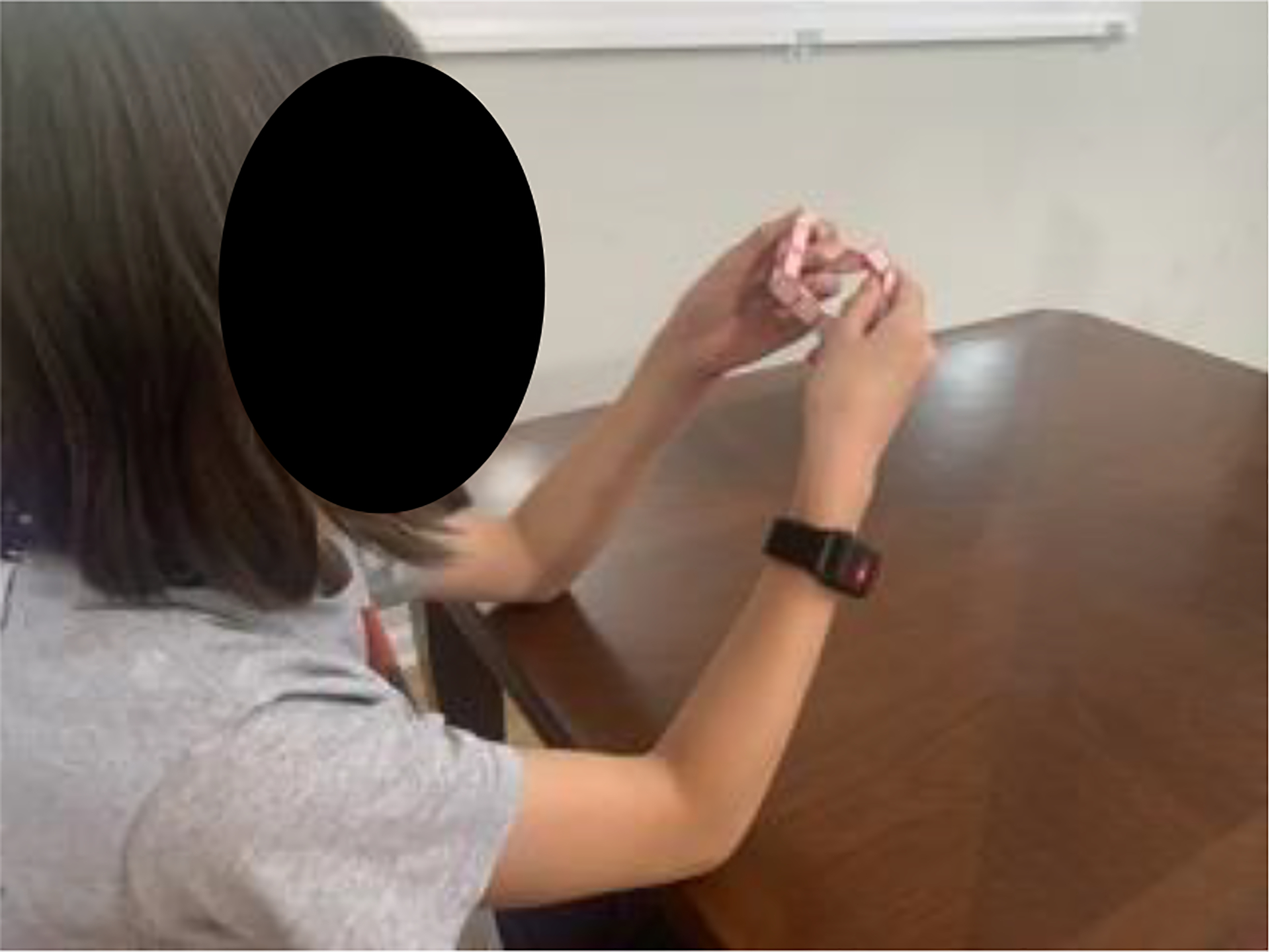
A picture of a nine-year-old neurotypical child using TheraBracelet during a game play shows the size of the wearable device.

**Figure 2.**
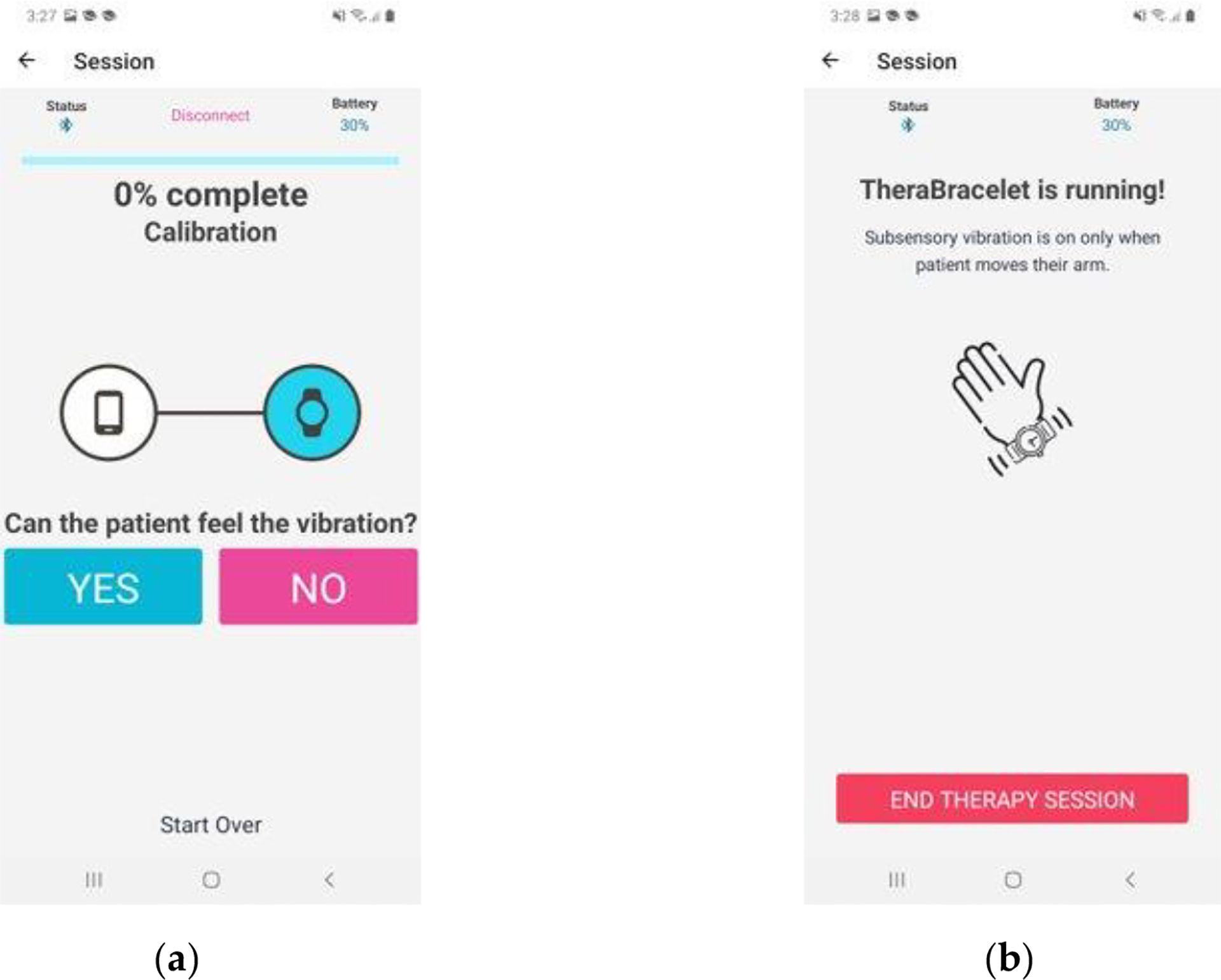
The smartphone application screen for calibration (A) and TheraBracelet stimulation delivery (B).

A custom-made, watch-like, wearable device (Figure 1) along with a smartphone containing a TheraBracelet smartphone application (referred to as App hereafter, Figure 2) was provided to the child. The child and parent were instructed to wear the device during the child’s daily activities and charge the device every night, while continuing with their regular activities and standard care therapy. No additional therapy service was provided, beyond what the child was already receiving prior to participating in this study. The child was instructed that the device was not waterproof and thus should not be worn during water activities. The child and parent were instructed to contact the study personnel for any questions.

The child and parent were instructed to use the TheraBracelet App by following the App prompts each time the child put on the wearable device. Once the App is activated, the smartphone automatically pairs with the wearable device using a Bluetooth connection and a calibration process begins. This calibration starts with the user answering a series of “yes” or “no” questions regarding whether the user feels a vibration from the device (Figure 2A). The App changes the level of vibration delivered by the wearable device based on the user’s responses until the user’s sensory threshold is determined as the lowest perceived vibration. Once the sensory threshold is determined, the App indicates that TheraBracelet stimulation is being delivered (Figure 2B).

The vibratory TheraBracelet stimulation is delivered in a random frequency at 60% of sensory threshold, which is considered imperceptible. The random frequency was based on the stochastic resonance literature and others showing the effect of temporarily non-uniform stimulation on the central nervous system and behaviors [44, 45, 47, 59–67]. The stimulation location and intensity parameter is used as it was found to be associated with improved hand function in previous studies [43, 50, 51]. TheraBracelet stimulation is provided only when movement of the affected upper extremity is detected by an accelerometer within the wearable device, as it is intended to amplify brain activity when the user is engaged in sensorimotor tasks [19, 20, 46, 47].

#### Intervention

We tracked one child’s experience of wearing the TheraBracelet for 1.5 years. This extended timeframe was chosen to allow the team to evaluate feasibility and sustained usability for a child. We wanted study findings to be able to include data on usage during the school year and over the summer. The child continued all life activities as usual (attending school, receiving standard outpatient therapy, routine medical interventions) providing insight into the various facilitators and barriers for incorporating wearing of the TheraBracelet into typical life.

#### Assessments

##### 1) Feasibility and usability.

The first marker of feasibility was simply the parent and child’s willingness to continue or drop out of the study. The parent also uploaded monthly self-report forms to a secure server in which the parent indicated the average hours per day the TheraBracelet was worn. In addition, any questions or problems expressed by the child and parent were documented. At the end of the study the child completed the System Usability Scale (SUS) [68, 69] to provide insight into the usability of the wearable device and the App.

##### 2) Safety.

The child’s physical therapist examined the child during therapy sessions and reported any adverse events related to the study participation to the study personnel via email approximately twice a month. In addition, any adverse events that the child and parent perceived were recorded in the monthly self-administered reports uploaded to the secure server.

##### 3) Upper limb motor function monitoring.

Several measures of upper limb motor function were obtained to detect deterioration in function, if any. These measures included the Goal Attainment Scale (GAS), ABILHAND-Kids, Bruininks-Oseretsky Test of Motor Proficiency Edition 2 (BOT-2) Upper Limb Coordination subtest, Box and Block Test, and Nine Hole Peg Test. The parent was instructed to administer these tests at home and record the results as part of the monthly self-administered report uploaded to the secure server. The parent was provided with online video resources for administration of these assessments. The parent and researchers also discussed these assessments and scoring rules. The parent videotaped the assessments and uploaded the videos to the server as well, and the accuracy of the assessments was confirmed by the research personnel. The parent was astute with impartiality and when unsure, asked the researchers to verify the scores.

- GAS uses a 5-level incremental scale from −2 to +2 (−2, −1, 0, +1, +2) for each goal at each time point. The child’s baseline ability is the base score of −2. The child’s “expected progress” is a score of 0. A score < 0 indicates “less than the expected goal” was achieved. A score of > 0 indicates “more than the expected goal” was achieved. Individual goal scores can be averaged to produce a cumulative score indicating overall intervention effectiveness [70].
- The ABILHAND-Kids assesses the parent’s perception of the level of difficulty that a child experiences when performing activities of daily living either bimanually or unimanually [71].
- The Upper Limb Coordination subtest of the BOT-2 assesses bilateral and unilateral upper extremity limb coordination, and scores can be reported as point scores and standard scores for each sub-test [72, 73].
- The Box and Block Test and the Nine Hole Peg Test assess the unilateral gross manual dexterity [74] and the digit dexterity [75], respectively.

## 3. Results

### Feasibility and usability

**Figure 3.**
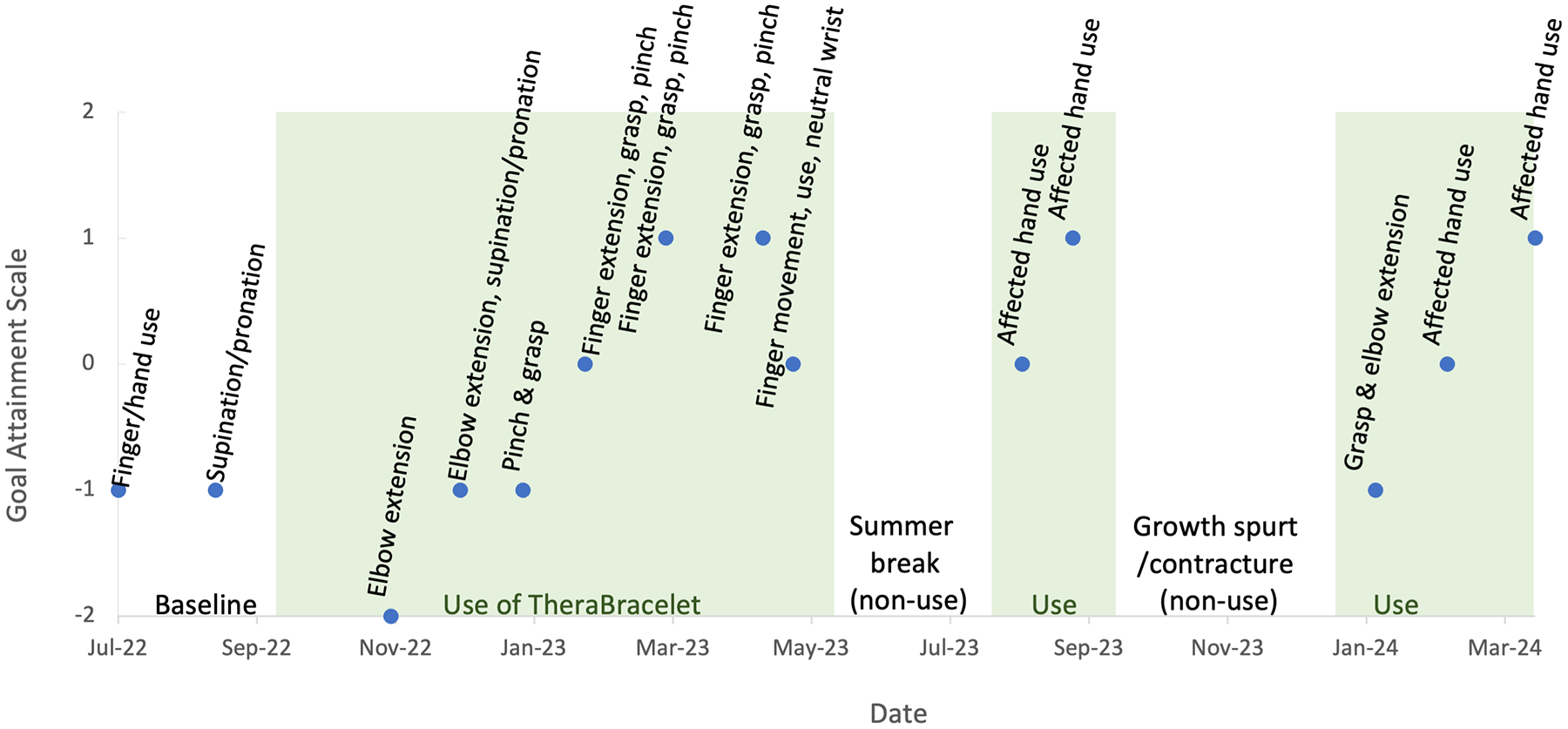
Goal attainment scale. The score of −2 and −1 indicate much less than expected and somewhat less than expected, respectively. The score of 0, 1, and 2 indicate expected, somewhat more than expected, and much more than expected. The shaded areas indicate periods of consistent TheraBracelet use. The non-shaded area at the beginning serves as the baseline prior to using TheraBracelet. The two non-shaded areas in the middle indicate the two breaks in TheraBracelet use. Although the parent was instructed to perform the assessments monthly, they could not complete the assessments every month. The dots represent the times at which the assessments were performed.

The child started using TheraBracelet in the fall of 2022 and continued the use except for two major breaks at the time of this report. The first break was over the summer of 2023. The child attended multiple summer camps and family beach vacations and did not use TheraBracelet due to water activities all day long. The second break in use occurred for 3 months in the fall of 2023 when the child had a growth spurt, resulting in an increased biceps contracture. The child had to wear an elbow extension orthotic from the elbow to proximal wrist crease which prevented the child from wearing the device. Otherwise, the child consistently used TheraBracelet as indicated in Figure 3. The child and parent continued the study without dropping out, and also expressed their willingness to continue using TheraBracelet beyond 2 years of the study duration.

The child primarily wore the device for approximately 3–4 hours every day after school and all day on weekends. The child used TheraBracelet at school only a few times. The child reported that he did not use the device at school consistently due to fear of losing the device or the smartphone. The child used the TheraBracelet while playing with friends indoors and outdoors, going shopping with his parents, attending church, and during all his occupational and physical therapy sessions.

The child and parent only contacted the study team for concerns two times during the study period. First, upon beginning use of TheraBracelet, the parent wanted to confirm that after calibration, the child was not supposed to feel the vibration. The study personnel confirmed that it was correct and added that TheraBracelet should be imperceptible as it should only boost natural sensorimotor signals, not override it. Second, the wearable device broke one time, but only after using TheraBracelet for over a year. When the child was unplugging the mini-USB charging cable from the wearable device, the top cover of the device fell off. The device was mailed to the research lab by the parent and was fixed and mailed back to the family in 2 weeks. No other issues with the device were reported.

In terms of usability, initially, the parent assisted with initial setup of the device using the on-screen directions provided in the App. After that, the child and parent reported that the child could don/doff and calibrate the wearable device independently. The parent also reported that the child could plug the device in to charge each night, needing only occasional assistance from a parent. The child’s SUS score was 87.5, indicating excellent usability [76, 77] of the device.

### Safety

There were no adverse events reported by the therapist, child, or the parent related to the participation in this study or use of TheraBracelet. The child’s physical therapist also did not report any adverse event or issues with the device during regular therapy sessions.

### Upper limb motor function monitoring

The GAS score improved over time. The goal attainment scores over time are shown in Figure 3. For the two initial months, the attainment of goals for finger/hand use and supination/pronation was less than expected. In 2023 and 2024, the attainment of goals such as finger extension, grasp, pinch, and increased use of the affected hand was more than expected.

**Figure 4.**
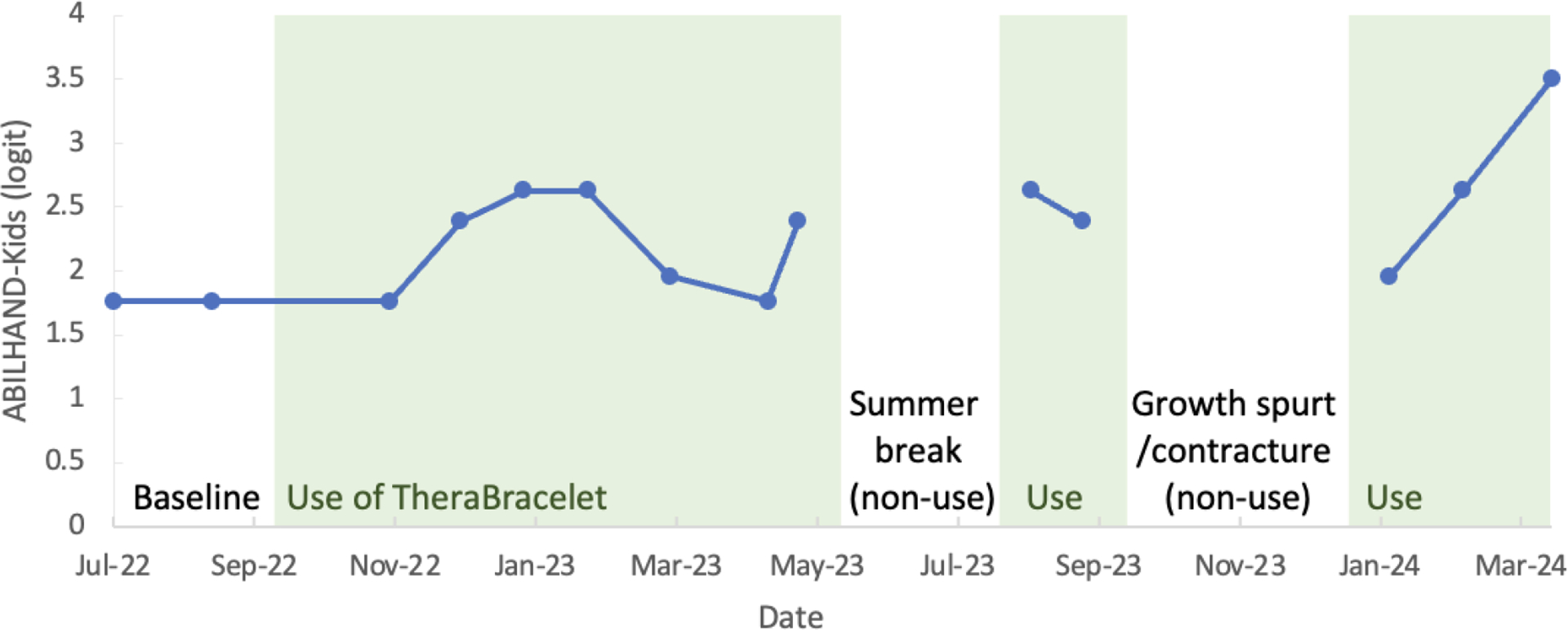
ABILHAND-Kids logit score.

The ABILHAND-Kids score converted to a logit score showed an increase over time (Figure 4). In addition, the parent noted details of how the task was performed for each activity. For instance, for zipping up trousers, the score worsened, and the parent’s note revealed that the child initially completed the task easily by using the unaffected hand only, and over time, the child started completing the task bimanually including the affected hand, but with difficulty. Similarly, for opening a jar or unscrewing a bottle cap, the child initially opened them with the unaffected hand while holding them with legs easily, and over time, the child started using the affected arm to hold but with difficulty. For zipping up a jacket and buttoning up a shirt and trousers, the child used to use adaptive tools such as magnetic zippers that allows one-handed zipping and a button hook. Over time, the child started performing the activities without adaptive tools using both hands. In summary, in addition to the ABILHAND-Kids score increase, there was a descriptive improvement in use of the affected hand for daily activities.

**Figure 5.**
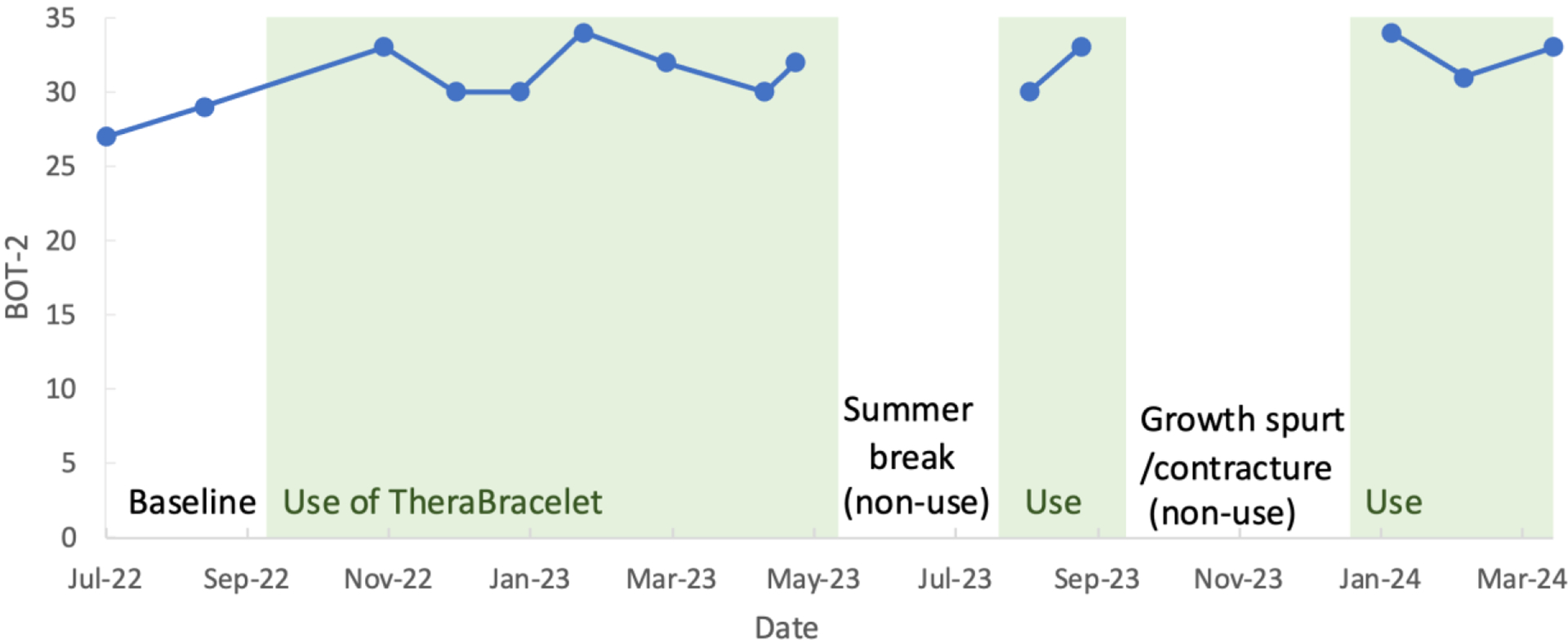
BOT-2 Upper Limb Coordination subtest total point score.

The BOT-2 Upper Limb Coordination subtest total point score showed a small increase over time (Figure 5). The improvement was primarily from two items related to bimanual coordination: catching a ball and dribbling a ball with alternate hands. The Box and Block Test score was 4 initially and did not show a consistent pattern of change. The child was not able to complete the Nine Hole Peg Test with his hemiplegic hand at any time during the study period.

## 4. Discussion

The case study was the first to investigate the feasibility of using TheraBracelet for a child with hemiplegic cerebral palsy in the home and community settings. The results of this study demonstrate that it is feasible for a child to use TheraBracelet during daily activities without adverse events. Motor function monitoring showed no sign of deterioration of motor function with prolonged use of TheraBracelet. These results encourage future research beyond a case report for use of TheraBracelet in children with cerebral palsy.

### Feasibility, usability, safety

The study examined feasibility in terms of retention, use extent, usability, and safety following guidelines [78, 79]. The child’s continued engagement and perceived useability of TheraBracelet occurred for several reasons. First, after initial assistance by the parent, the child had enough cognitive and motor ability to charge the devices, don and doff the wearable device by himself, perform calibration with the smartphone App, and use TheraBracelet independently. Second, the TheraBracelet device was sturdy and only required minimal repair after a year and a half of wear. Third, the high SUS score indicating excellent usability [77] is consistent with a previous finding that demonstrates use of the wearable device and smartphone App was easily achievable [80]. The perceived utility and ease of use may explain the child and parent’s willingness to continue using TheraBracelet, even beyond 2 years of the study duration and support adoption of TheraBracelet for wider usage in the future [81].

### Upper limb motor function

Monitoring of upper limb motor function showed no sign of deterioration. The child met GAS goals relate to improved finger extension, pinch, grasp, and increased use of the affected hand. Based on the ABILHAND-Kids, perceived difficulty in completing activities of daily living improved, along with use of the affected upper extremity in bimanual activities such as buttoning up clothes and opening a bottle. While information on the minimally clinically important difference (MCID) for the ABILHAND-Kids is unavailable, MCID for chronic stroke survivors was found to be 0.26 to 0.35 logits [82]. Over the period examined, the child experienced a 1.742 logit score increase, which was deemed meaningful by the parent. In addition, the child’s BOT-2 Upper Limb Coordination subtest total point score increased by 6, with improved abilities to catch a tossed ball with both hands and dribble a ball with alternating hands. While the objective measure of BOT-2 shared the similar trend with subjective measures (e.g., ABILHAND-Kids), other objective measures of the Box and Block Test and the Nine Hole Peg Test did not change consistently. Given that the child could not place any peg to a hole for the Nine Hole Peg Test throughout the study duration, this assessment was too high level and not a good choice of functional assessment for this child. It is also possible that the Box and Block Test may not be as motivating as BOT-2 with a ball play for this child. The present study may inform the choice of adequate assessments for future studies. Overall, this study found improvements in the hand-object manipulation skills. Improvements in hand-object manipulation skills are consistent with the previous reports that TheraBracelet led to improvements in grasping skills more so than reaching in adult stroke survivors [55].

### Limitations

While the strength of this study is an extended study duration of 2 years with daily procedures to investigate the long-term influence of TheraBracelet on a child with cerebral palsy, the major limitation is that this study is a case report involving only one child. A case report provides the lowest level of evidence and is a starting point toward more controlled studies [83]. The results of this case report are not generalizable to other children with cerebral palsy. The changes noted in achievement of goals and improvements in motor function are encouraging, especially the affected hand use in daily activities, when previous in-lab-only interventions resulted in the improved upper limb movement capacity but no increase in use of the affected upper limb in daily living [84, 85]. These findings highlight the potential benefits of using TheraBracelet during daily activities as an adjunct to therapy in the home and community settings. However, we cannot exclude the possibility that these improvements were due to the child’s maturation over the time he participated in the study, his ongoing weekly therapy, or learning from repeated administration of assessments. Therefore, the results of this study should be interpreted with caution. Future research must include more children and a control group to ascertain TheraBracelet’s efficacy. Specially designed therapy focused on improving hand-object manipulation abilities may be paired with TheraBracelet to draw out the benefit of TheraBracelet. Future studies should also stratify for age, gender, functional level including sensory impairment level [86], and therapy dosage and activities to characterize responses. While the hand-object manipulation function is the targeted outcome based on adult trials [36–38, 55], future studies may explore outcomes in more domains such as participation and activities [87] that are appropriate for developmental milestones.

One factor that limited use of TheraBracelet in this study was that the device was not waterproof. Further developing a waterproof wearable device may help with continued use of TheraBracelet in children, especially for wear during summer months when children increase participation in water leisure activities. The parent mentioned that if she could monitor her son’s TheraBracelet use on her own smartphone (as opposed to only on the child’s smartphone), she could ensure and encourage the use. Thus, further development for use monitoring via cloud server for parents and researchers could be beneficial. Such development may be accompanied with monitoring of the affected hand use extent using accelerometry [84] or machine learning-based movement quality classification [88–90] for greater utility.

## 5. Conclusions

This case report shows that it was feasible for a child with cerebral palsy to use TheraBracelet in daily living without adverse events and with improved affected hand movement and bimanual activity. However, this study is only a case report, and a larger trial is recommended to determine the efficacy of TheraBracelet with this population. The results of this study encourage future research for use of TheraBracelet as a beneficial therapy adjuvant to improve the affected upper extremity function and use in daily living in children with cerebral palsy.

## 6. Patents

There is a patent regarding the vibrotactile stimulation U.S. Patent No. US 10,071,015 B2.

## Data Availability

All data are presented within the manuscript.
